# Proof-of-Concept Quantum Simulator Based on Molecular Spin Qudits

**DOI:** 10.1021/jacs.3c12008

**Published:** 2023-12-26

**Authors:** Simone Chicco, Giuseppe Allodi, Alessandro Chiesa, Elena Garlatti, Christian D. Buch, Paolo Santini, Roberto De Renzi, Stergios Piligkos, Stefano Carretta

**Affiliations:** † Dipartimento di Scienze Matematiche, Fisiche e Informatiche, Università di Parma, I-43124 Parma, Italy; ‡ INSTM, UdR Parma, I-43124 Parma, Italy; § INFN-Sezione Milano-Bicocca, Gruppo Collegato di Parma, I-43124 Parma, Italy; ∥ Department of Chemistry, University of Copenhagen, DK-2100 Copenhagen, Denmark

## Abstract

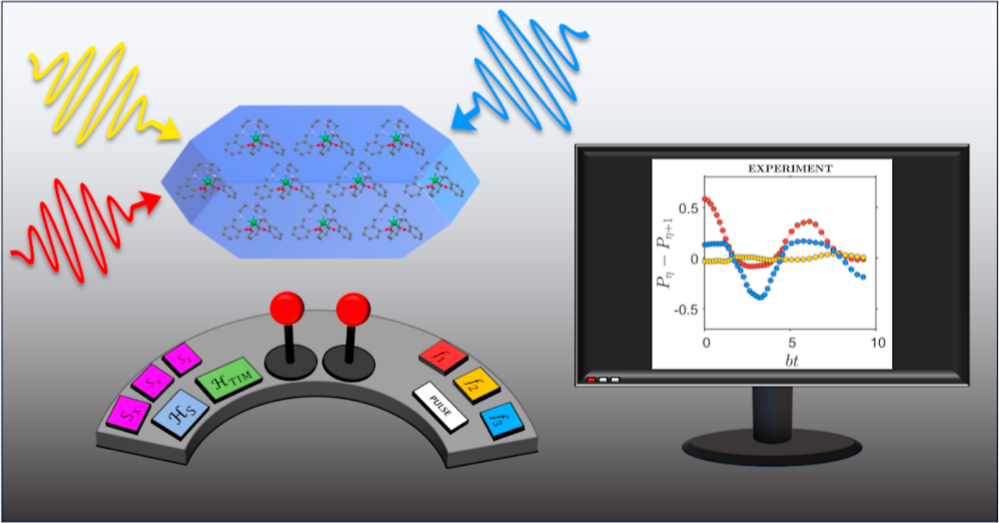

The use of *d*-level qudits instead of two-level qubits can largely increase the power of quantum logic for many applications, ranging from quantum simulations to quantum error correction. Magnetic molecules are ideal spin systems to realize these large-dimensional qudits. Indeed, their Hamiltonian can be engineered to an unparalleled extent and can yield a spectrum with many low-energy states. In particular, in the past decade, intense theoretical, experimental, and synthesis efforts have been devoted to develop quantum simulators based on molecular qubits and qudits. However, this remarkable potential is practically unexpressed, because no quantum simulation has ever been experimentally demonstrated with these systems. Here, we show the first prototype quantum simulator based on an ensemble of molecular qudits and a radiofrequency broadband spectrometer. To demonstrate the operativity of the device, we have simulated quantum tunneling of the magnetization and the transverse-field Ising model, representative of two different classes of problems. These results represent an important step toward the actual use of molecular spin qudits in quantum technologies.

## Introduction

Magnetic molecules, whose magnetic core is typically made of one or few exchange coupled magnetic ions, have provided an ideal playground to investigate fundamental phenomena, ranging from quantum tunneling of the magnetization in isolated molecules^1,2^ to hysteresis at 60–80 K of single-molecule origin^3,4^ or decoherence.^5,6^ A strength point of this class of materials is that their complex single-molecule spin dynamics can be accessed even by bulk measurements.^7,8^ Nevertheless, coherent manipulation and readout of a single TbPc_2_ molecule was shown in a single-molecule transistor.^9,10^

Being controllable quantum objects, magnetic molecules have attracted considerable attention as molecular qubits,^11−13^ thanks to the remarkable possibilities of engineering their Hamiltonian^14^ and the long coherence times (from hundreds of μs to ms) reported in Cu^15^ or VO complexes.^16−18^ Moreover, the possibility of controlling their quantum state by electric fields^19−22^ and the blueprint of a magnetic quantum processor^23^ have been recently shown. These results are very interesting, but what makes magnetic molecules really potentially disruptive for quantum technologies is the fact that they naturally provide multilevel quantum systems, i.e., qudits with large number of states.^24−27^ Indeed, the use of qudits as elementary units of computation^28−31^ can simplify or improve quantum algorithms^32−37^ and quantum sensing protocols.^38^ For instance, qudit encoding can significantly reduce the number of two-body gates and thus improve the implementation of quantum algorithms.^37,39^ Moreover, by enconding a protected qubit into a single multilevel object, quantum error correction could be implemented without the large overhead of resources required by qubit-based codes.^25,40−44^

In the past decade, many efforts have been focused on using molecular qudits as quantum simulators (QSs).^45−49^ QSs are controllable quantum systems whose dynamics is externally driven to calculate the ground state^50^ or to mimic the time evolution^51^ of the “target” Hamiltonian, i.e., the Hamiltonian of the model that needs to be simulated. QSs made of molecular qudits would be very interesting, because problems involving quantum objects with many degrees of freedom can be solved more efficiently by going beyond the binary qubit logic. For instance, nuclear^52^ or bosonic^48^ Hamiltonians can be naturally mapped to the higher dimensional qudit Hilbert space, avoiding the large growth of qubits^53^ or complex gates^54^ typical of multiqubit encodings. Moreover, a QS based on molecular qudits could embed quantum error correction. However, in spite of more than a decade of efforts, an experimental realization of a QS based on MQs was still lacking, thus leaving their striking potential completely unexpressed.^13^

Here, we show the first realization of a working proof-of-concept quantum simulator based on an ensemble of ^173^Yb(trensal) qudits,^55^ and we demonstrate its operation by implementing the quantum simulation of models representative of two different classes of problems: an integer spin >1/2 subject to quantum tunneling of the magnetization (QTM) and a pair of spins 1/2 coupled by Ising interaction in the presence of a transverse field (transverse field Ising model, TIM). In both cases, our QS reproduces the correct physical behavior, and the results are in good agreement with calculations.

## Results and Discussion

### Quantum Hardware

**Figure 1:**
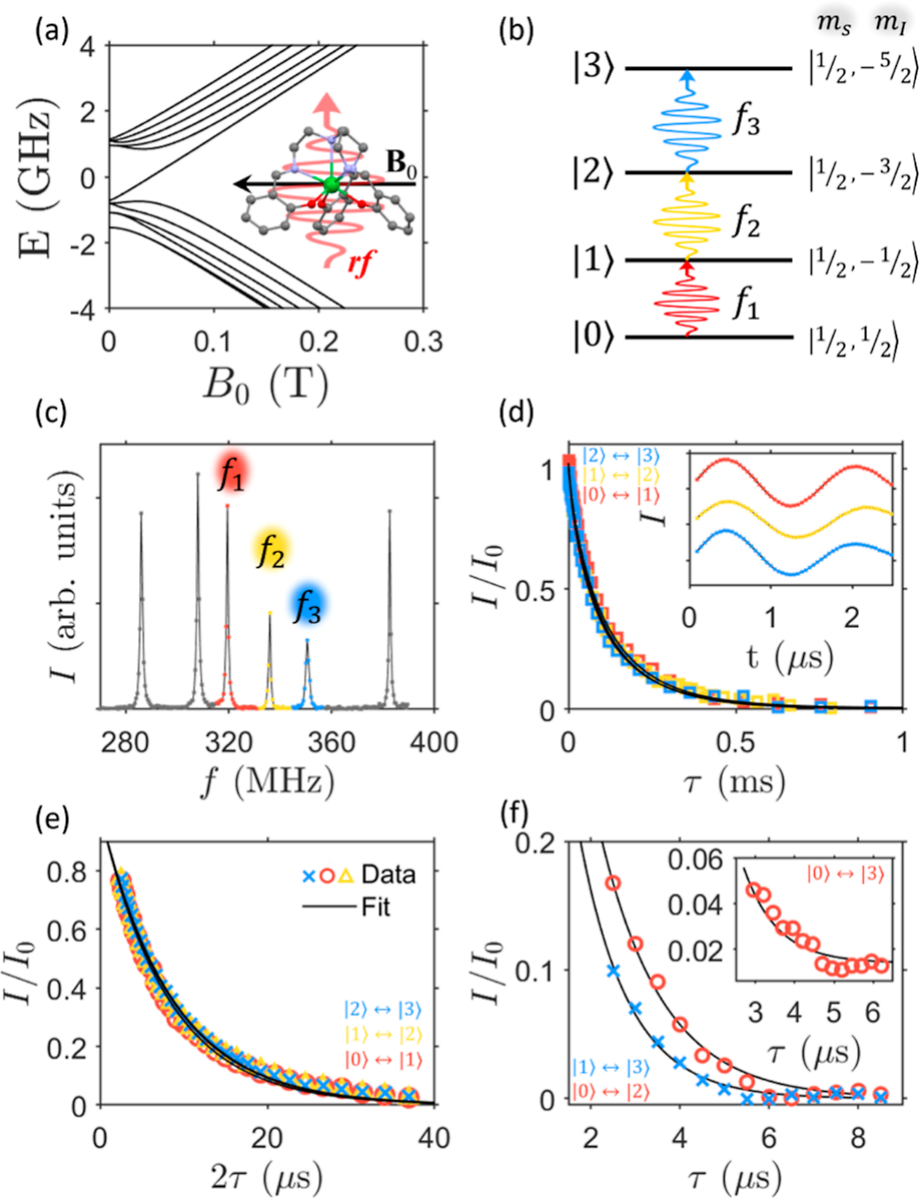
Calibration of the quantum hardware. (a) Calculated energy level diagram of ^173^Yb(trensal) with the static field **B**_0_ perpendicular to the molecular *C*_3_ axis. The molecule and the direction of the static (black) and driving (red) fields are shown as inset. (b) Scheme of the nuclear qudit subspace targeted in this work, with states labeled as |0⟩, |1⟩, |2⟩, and |3⟩ and transition frequencies as *f*_1_, *f*_2_, and *f*_3_ in the ascending order. (c) Example of the NMR spectrum of the ^173^Yb(trensal) qudit at *B*_0_ = 0.12 T and *T* = 1.4 K, with the peaks representing the nuclear transitions within the computational subspace highlighted in colors. (d) Relaxation times *T*_1_^η^ measured (dots) on each of the nuclear transitions with the multifrequency protocol, with *B*_0_ = 0.22 T and *T* = 1.4 K. Inset: some examples of coherent Rabi manipulation of the transitions indicated in panel (b) (labeled in color-code), demonstrating universal qudit control. (e) Phase memory time *T*_2_^η^ measured for each transition marked in panel (c), at *B*_0_ = 0.22 T and *T* = 1.4 K. (f) Double (main) and triple (inset) quantum coherence times. Error bars are within the size of the symbols.

The core of the quantum simulator consists of a crystal containing isotopically enriched ^173^Yb(trensal), doped at 1% into its diamagnetic [Lu(trensal)] isostructural analogue (see Experimental Section). Due to the large crystal field splitting of Yb(III), each molecule behaves as an electronic spin qubit (effective spin *S* = 1/2) coupled to a 6-levels nuclear spin qudit *I* = 5/2, providing 2 × 6 states. The corresponding spin Hamiltonian is given by
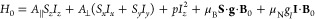
1where the first two terms represent the strong axial hyperfine interaction (*A*_∥_ = −898 MHz, *A*_⊥_ = −615 MHz), the third one describes the nuclear quadrupolar coupling (*p* = −66 MHz) and the last two are the electronic (*g*_*x*_ = *g*_*y*_ = 2.9, *g*_*z*_ = 4.3) and nuclear (*g*_*I*_ = −0.2592) Zeeman terms. These parameters, determined in previous works^55,56^ (see Supporting Information, Figure S1), provide sufficient energy separation of the nuclear transitions for the selective manipulation of each energy gap, while being close enough for addressing multiple transitions within our broad multifrequency setup. Static fields *B*_0_ between 0.12 and 0.22 T are applied orthogonal to the molecular *C*_3_ symmetry axis (Figure 1a). At these fields, the electronic Zeeman energy is the leading term in eq 1, thus the eigenstates are almost factorized and are labeled by the dominant electronic and nuclear spin components along **B**_0_, |*m*_*S*_, *m*_*I*_⟩. Here, we focus on states |*m*_*S*_ = 1/2, *m*_*I*_⟩, with *m*_*I*_ = 1/2, −1/2, −3/2, −5/2 and use the simplified notation |0⟩, |1⟩, |2⟩, |3⟩, as shown in Figure 1b. The corresponding transition frequencies are *f*_1_ (|0⟩ ↔ |1⟩, red), *f*_2_ (|1⟩ ↔ |2⟩, yellow), and *f*_3_ (|2⟩ ↔ |3⟩, blue).

The use of an ordered ensemble of identical qudits as QS has the advantage of yielding the expectation values with high statistics directly in a single run. Full control of the qudits is achieved by addressing each energy gap using a flexible broadband NMR spectrometer equipped with a tailored multifrequency probe spanning the frequency range ∼200–450 MHz. The driving Hamiltonian is
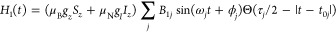
2where Θ is the Heaviside step function and the sum runs over different pulses of amplitude *B*_1*j*_ (parallel to the *c* axis), duration τ_*j*_, center *t*_0*j*_, frequency ω_*j*_/2π, and phase ϕ_*j*_ addressing consecutive (Δ*m*_*I*_ = ±1) transitions (i.e., ω_*j*_ = *f*_η_ × 2π, with η = 1, 2, 3). The simulator operates at 1.4 K, a temperature at which all the eigenstates are populated. Hence, we prepare an initial pseudopure state by proper sequences of pulses (see subsection on Quantum Tunneling and Experimental Section).

### Calibration

We first need to show that a universal set of gates can be implemented in the QS and calibrate it. The NMR spectrum is reported in Figure 1c, with the transition frequencies *f*_1_ = 319.5 MHz, *f*_2_ = 336.0 MHz, and *f*_3_ = 350.5 MHz at *B*_0_ = 0.12 T (*f*_1_ = 333.7 MHz, *f*_2_ = 362.4 MHz, and *f*_3_ = 386.2 MHz at *B*_0_ = 0.22 T, see Supporting Information, Figure S1), highlighted in the corresponding color code. ^173^Yb(trensal) has sharp spectral lines (fwhm ∼ 0.5 MHz), ensuring the possibility of individually addressing the transitions (see Figures 1b and S2, Supporting Information). To demonstrate full coherent control, we performed transient nutation experiments to induce Δ*m*_*I*_ = ±1 Rabi oscillations with arbitrary phases between all the selected nuclear states (inset of Figure 1d). These operations are the basic gates building up our quantum simulation sequences. Rabi experiments were also exploited to set the duration of the pulses to be nearly the same, by calibrating the driving fields used for the QS (see Supporting Information, Table S1).

Relaxation times much longer than the time needed to perform the full gate sequence and sufficiently long coherence times are required to perform a reliable quantum simulation. Thus, we measured all the relevant characteristic times *T*_1_^η^ and *T*_2_^η^ in the experimental conditions exploited in the quantum simulations. First, the relaxation times *T*_1_^η^ of the three selected transitions were probed by exploiting a double-frequency method. The signal decay is profiled by probing the transition *f*_η_ between states |η – 1⟩ and |η⟩ after an out-of-equilibrium surplus population is induced by an excitation pulse on the transition *f*_η±1_, to investigate the relaxation toward thermal equilibrium of diagonal elements of the density matrix (see Experimental Section). The results obtained at the applied static field *B*_0_ = 0.22 T are reported in Figure 1d, yielding *T*_1_^η^ values of the order of 100 μs for all the transitions. Similar results were obtained at *B*_0_ = 0.12 T (see Supporting Information, Figure S3).

Single-quantum coherence times *T*_2_^η^ (of superpositions between states with Δ*m*_*I*_ = 1) were measured by a standard Hahn-echo pulse sequence and are shown in Figure 1e (see also Supporting Information, Figure S4). The three transitions *f*_η_ (η = 1, 2, and 3) show very similar *T*_2_^η^ ∼ 8 μs, significantly longer than simulation times. Additional key pieces of information for qudit-based architectures are the coherence times of superpositions involving Δ*m*_*I*_ > 1 states, the so-called multiple-quantum coherences. These superpositions are in fact created during quantum simulations, and their characterization is therefore important for the design of optimized sequences. In order to extract multiple-quantum coherences, we first created the desired Δ*m*_*I*_ > 1 superposition exploiting π-pulses for state swaps (see Experimental Section). After a variable delay, we used π pulses to back swap the states and employ a  sequence for detecting the decay of these coherences. Results for double- and triple-quantum coherences between the selected nuclear states are reported in Figure 1f (main panel and inset, respectively). Since multiple-quantum superpositions involve states which are magnetically more different from each other, we found shorter coherence times with respect to single coherences (∼1.2 μs for Δ*m*_*I*_ = 2 and ∼0.7 μs for Δ*m*_*I*_ = 3). However, because of the chosen encoding, the system is placed into superpositions of multiple states only for time intervals much shorter than the full sequence duration. Thus, these coherences are shown not to affect significantly the quantum simulation (see Supporting Information, Figure S5).

As shown later in the article, these values permit the QS to capture the physics of the target models.

### Quantum Simulations

The versatility of the QS is demonstrated by performing two different quantum simulations exploiting the multilevel structure of the molecular qudit: (i) the quantum tunneling of the magnetization of a single *S* = 1 spin, where the 2*S* + 1 states of the target system are mapped onto the hardware levels and the unitary evolution is exactly decomposed into transitions between neighboring levels (see subsection on Quantum Tunneling). (ii) The time dependence of the magnetization and of the correlation function for two spins 1/2 in a transverse magnetic field in two different regimes: either noninteracting or with an Ising coupling. Here, the two-spin Hilbert space is mapped onto the single qudit energy levels and the unitary evolution induced by the target Hamiltonian is decomposed into a sequence of elementary steps by using the Suzuki–Trotter approximation. This explores the possibility of encoding several spins into single qudits (see subsection on the Transverse Field Ising Model).

#### Quantum Tunneling

**Figure 2:**
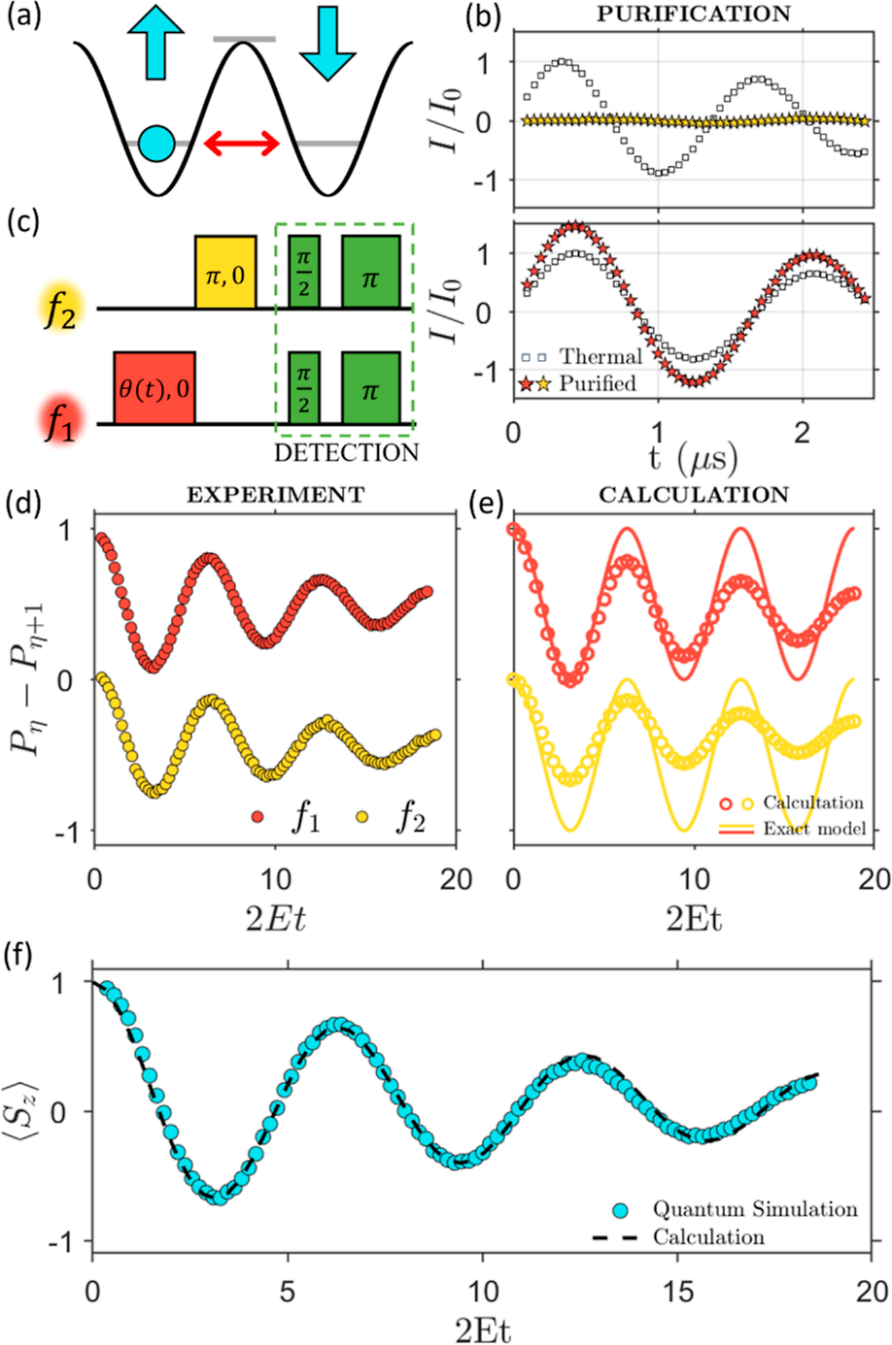
Simulation of quantum tunneling of the magnetization. (a) Sketch of the double-well axial crystal field potential acting on a spin *S* = 1 system prepared in *M* = 1 (circle) and subject to quantum tunneling activated by rhombic anisotropic terms (red double-arrow). (b) Test of the purification protocol by sending pulses at frequency *f*_2_ (top) and *f*_1_ (bottom), respectively addressing |1⟩ ↔ |2⟩ and |0⟩ ↔ |1⟩ transitions, and comparing the driven dynamics before and after purification. (c) 2-Frequency pulse sequence consisting of a pulse of length θ(*t*) = *Et* at frequency *f*_1_, followed by a π pulse at frequency *f*_2_ and concluded by Hahn-echo detection. (d) Difference of populations between consecutive levels |0⟩ ↔ |1⟩ (red) and |1⟩ ↔ |2⟩ (yellow), measured at *B*_0_ = 0.12 T and *T* = 1.4 K by Hahn-echo sequences at frequencies *f*_1_ and *f*_2_, respectively, at the end of the quantum simulation. (e) Corresponding noiseless calculations (lines) or including measured single- and double-quantum *T*_2_^η^, as well as additional dephasing due to inhomogeneities of the driving field (circles). (f) Measured (blue circles) and calculated (dashed line) expectation value of the magnetization of the target system. Error bars are within the size of the symbols.

We consider an *S* = 1 target system characterized by the Hamiltonian (with *D* > 0)
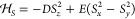
3For *E* = 0, this corresponds to the double-well potential sketched in Figure 2a, where the ground state is a degenerate doublet with maximum absolute value of the magnetization (arrows in Figure 2a), i.e., *M* = ±*S*. A small rhombic anisotropy term *E* in  activates quantum tunneling through the barrier and hence a system prepared in one of the two wells oscillates between states with opposite magnetization.

To simulate the phenomenon, the three levels of the *S* = 1 target system are mapped onto the hardware states |0⟩, |1⟩, and |2⟩ of Figure 1b, which are initially in a thermal mixture because our experiment is not at *T* = 0. Therefore, we prepare the initial pseudopure state in this subspace by first applying a π/2 pulse at frequency *f*_2_ which creates a superposition between states |1⟩ and |2⟩ with equal amplitudes. This is followed by a waiting time ∼2.5*T*_2_^2^ to let the relative coherence decay. The resulting density matrix in the {|0⟩, |1⟩, |2⟩} subspace is therefore of the form ρ_0–2_ = ϵ|0⟩⟨0| + (*p*_1_ + *p*_2_)/2(|0⟩⟨0| + |1⟩⟨1| + |2⟩⟨2|), with ϵ = *p*_0_ – (*p*_1_ + *p*_2_)/2 and *p*_η_ the initial Boltzmann population of the energy states. Apart from normalization, this state is equivalent for quantum simulation to the *pure* density matrix ρ_0–2_ = |0⟩⟨0|. Indeed, the part of ρ_0–2_ proportional to the identity in the considered subspace does not produce any signal in our experiment. To check the “purification” procedure, we compare in Figure 2b Rabi oscillations addressing transitions |1⟩ ↔ |2⟩ (top) and |0⟩ ↔ |1⟩ (bottom) before and after the sequence. Without purification (i.e., with thermal populations), a pulse of variable length at frequency *f*_2_ induces oscillations between states |1⟩ and |2⟩. Conversely, after the purification sequence states |1⟩ and |2⟩ start with equal populations and hence Rabi oscillations are not observed (Figure 2b, top), as it would occur at *T* = 0. Concerning the transition |0⟩ ↔ |1⟩, the purification protocol enhances by about 50% their population difference, resulting in an amplification of Rabi oscillations (Figure 2b, bottom). In addition, we verified that coherences are lost after the waiting time (see Experimental Section and Supporting Information, Figure S6).

Having tested that the prepared state is spectroscopically equivalent to the pure state |0⟩, we illustrate the simulation of the tunneling dynamics as a function of the simulation time *t*. The optimized sequence^57^ is shown in Figure 2c, ending with Hahn echo sequences at frequencies *f*_1_ and *f*_2_ to access differences between the populations of neighboring levels of the hardware *P*_η_ – *P*_η+1_.

Results are shown in Figure 2d, in excellent agreement with calculations (Figure 2e) including decoherence in Lindblad formalism (see Experimental Section) and an additional decay ascribed to inhomogeneity of the driving field.^55,58^ From these population differences we can extract the target observable ⟨*S*_*z*_⟩ = *P*_0_ – *P*_2_, i.e., the magnetization of the simulated system (see Figure 2f). This displays the expected quantum oscillation at frequency *E*/π, in very good agreement with calculations.

#### Transverse Field Ising Model

We now consider a different problem, represented by a target system of two spins 1/2, interacting via the Hamiltonian
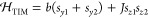
4where *s*_α*i*_ are spin 1/2 operators and we set *b* = *J*. The quantum simulation of the corresponding time evolution  requires to decompose *U*(*t*) into elementary operations which can be implemented on the hardware. In most qubit-based processors, this implies separately simulating one- and two-body terms in eq 4 and then applying a Suzuki–Trotter (ST) approximation to *U*(*t*), i.e.
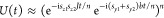
5Such an approximation becomes exact for a large number of Trotter steps *n*, at the price of an increasing number of noisy gates. Nevertheless, a proper trade-off can be found to reproduce the correct dynamics at not too large simulated times with a rather small *n*, thus limiting decoherence.

**Figure 3:**
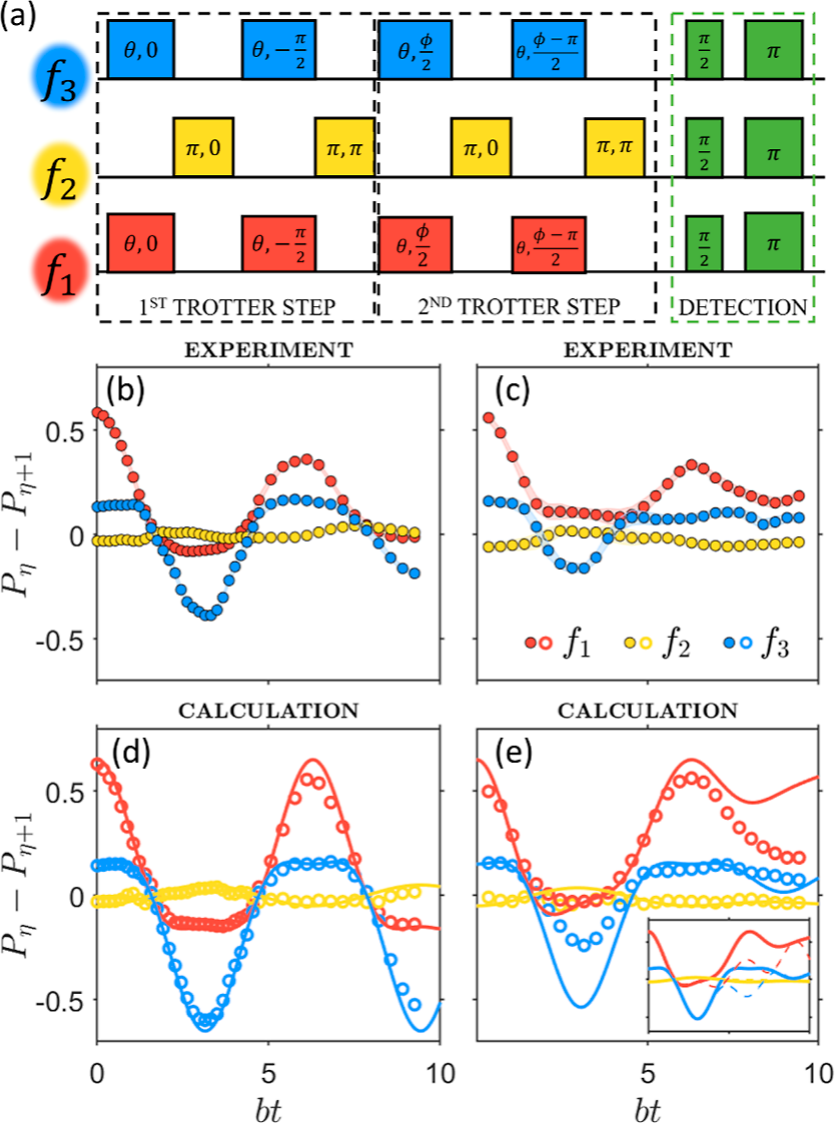
Simulation of the transverse Ising model. (a) 3-Frequency pulse sequence to implement the quantum simulation of the transverse-field Ising model on 4 levels of the hardware qudit and to detect the final output. (b,c) Difference of populations between neighboring levels, measured at *B*_0_ = 0.22 T and *T* = 1.4 K by echo-sequences at the three driving frequencies *f*_1_ (red), *f*_2_ (yellow), and *f*_3_ (blue) for the non-interacting (b) and interacting (c) cases. The shaded areas represent the estimated experimental uncertainties in the amplitude determination. (d,e) Corresponding calculations for *n* = 2 with the inclusion of the incoherent Lindblad dynamics induced by the measured single-, double-, and triple-quantum coherence times. Inset of panel (e): results for *n* = 2 Suzuki–Trotter decomposition compared with the exact evolution induced by the target Hamiltonian (dashed lines).

Here, the four states of the target two-spin system {|↑↑⟩, |↑↓⟩, |↓↑⟩, |↓↓⟩} are mapped onto the qudit subspace {|0⟩, |1⟩, |2⟩, |3⟩}. Hence, each one-body unitary gate in eq 5 is simulated by a pair of pulses of the same length θ = *bt*/*n* at frequencies *f*_1_ and *f*_3_, simultaneously addressing |0⟩ ↔ |1⟩ and |2⟩ ↔ |3⟩ transitions. This directly implements a rotation of the second qubit, i.e., exp[−i*s*_*y*2_*bt*/*n*].^59^ The same pulses, preceded and followed by a π state-swap at frequency *f*_2_, implement a rotation of the first qubit exp[−i*s*_*y*1_*bt*/*n*]. The resulting sequence yields the exact quantum simulation of  for the noninteracting (*J* = 0) case, and it also corresponds to the first Trotter step of the interacting case (Figure 3a, left). The simulation of the two-body term exp[−i*s*_*z*1_*s*_*z*2_*Jt*] on a qubit hardware would require controlled-phase gates at the end of each Trotter step. In our qudit architecture, this entangling error-prone gate can be rewritten in terms of single-qudit operations, simply adjusting the phases of the pulses addressing consecutive |0⟩ ↔ |1⟩ and |2⟩ ↔ |3⟩ transitions, as shown in Figure 3a (for the second Trotter step).

An extension of the purification protocol illustrated above is used also in this second experiment to prepare the initial state (see Experimental Section and Supporting Information, Figures S7 and S8). Detection of the output state is accomplished again by Hahn echo sequences at the frequencies *f*_1_, *f*_2_ and *f*_3_.

**Figure 4:**
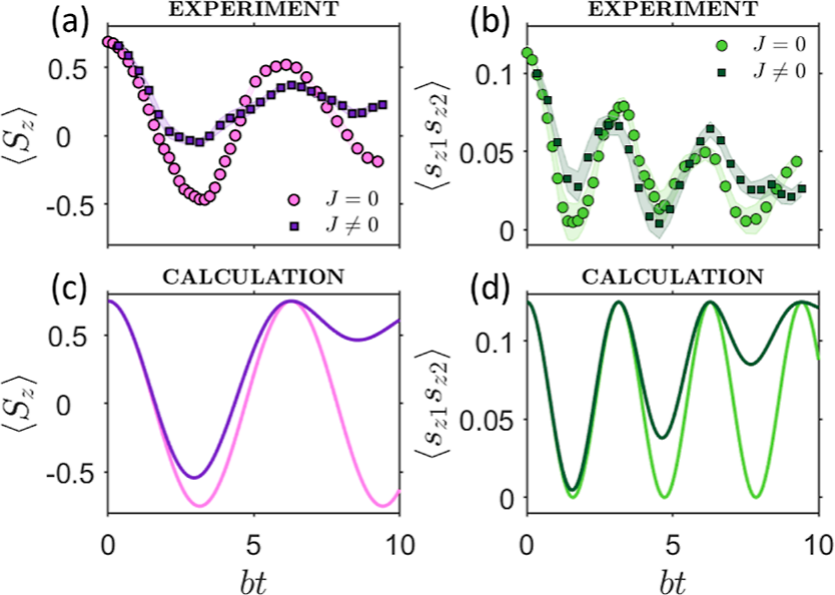
Observables for the transverse-field Ising model. Comparison between (a) total magnetization *S*_*z*_ = *s*_*z*1_ + *s*_*z*2_ and (b) equal-time cross-correlation function ⟨*s*_*z*1_*s*_*z*2_⟩ for the examined two-spin model without (*J* = 0) and with (*J* = *b*) Ising spin–spin coupling. Error bars represent the estimated uncertainties propagated from the experimental amplitudes of Figure 3b,c. They are more important for ⟨*s*_*z*1_*s*_*z*2_⟩, where the signal results from a subtraction of experimental data. (c,d) Corresponding noiseless calculations (lines) for *n* = 2.

Population differences measured at the end of the quantum simulation are reported in Figure 3 in noninteracting (b) and interacting (c) regimes, while corresponding observables are shown in Figure 4. Whereas for *J* = 0 the simulation is exact, for *J* ≠ 0, two Trotter steps are sufficient to capture the dynamics for *bt* ≲ 5 (inset of Figure 3e). Nevertheless, we have explored also longer simulation times to make a more stringent demonstration of our capability of controlling the quantum hardware in the presence of the complex dynamics induced by this sequence. Several of the pulses for the *J* ≠ 0 case have been applied in parallel (Figure 3a) to make the duration of the sequences similar in the two cases and hence less dependent on decoherence. The simulation could be extended to longer times by an exact decomposition in planar rotations, which however requires a significantly longer pulse sequence.

From Figure 3b–e, we note a good agreement between experimental results (b,c) and calculations for *n* = 2 (d,e), where the measured coherence times are included in a Lindblad formalism (circles). Pure dephasing here induces a damping of the oscillations of *P*_η_ – *P*_η+1_ (dashed lines), but the nontrivial time dependence induced by the target Hamiltonian is well reproduced. Hence, our quantum simulator is able to catch the correct physical behavior of the target system. In particular, the total magnetization *S*_*z*_ = *s*_*z*1_ + *s*_*z*2_ and the equal-time correlation ⟨*s*_*z*1_*s*_*z*2_⟩ simulated by the QS are reported in Figure 4a,b and compared with exact calculations for *n* = 2 (c,d). The QS predicts the oscillation frequency to be larger in the correlation than in the total magnetization, in good agreement with calculations. This agreement is remarkable especially for correlations, which are difficult to simulate because they are obtained from the difference of measured quantities (see Experimental Section). In addition, the differences in the time-dependence between the interacting and noninteracting cases in the magnetization are captured by the QS.

## Conclusions and Perspectives

We have demonstrated a proof-of-concept quantum hardware which explicitly makes use of the multilevel structure of molecular qudits as a key resource for quantum simulation. This is done by following two different approaches, targeting different classes of problems:

(1) the dynamics of a single multilevel system is directly mapped onto the energy levels of the qudit. This scheme can be extended from *S* > 1/2 problems to bosonic or Fermionic degrees of freedom, which are of crucial interest but require complex encodings on multiqubit platforms.^48,52−54^
(2) we have considered a multispin system whose Hilbert space is encoded into a single-qudit.^13^ This approach is important for the scalability of the platform in the near future. By encoding several spins of the target Hamiltonian into the same qudit, we significantly reduce the number of two-body gates, which are usually the most error-prone operations. Then, one can exploit a register consisting of several molecular (nuclear) qudits interacting via their electronic spins,^47^ to implement gates between different qudits. This can be still done in an ordered ensemble like a magnetically diluted crystal.

To further increase the scalability, the electronic spins can be used to activate an effective communication between distant qudits mediated by photons in superconducting resonators,^23^ after having swapped quantum information from the nuclear spins. This is made possible by the specific choice of molecular qudits as elementary units.

The presence of metal ions whose spins are strongly coupled to nuclear ones provides specific features which make this architecture different from standard liquid-state NMR quantum computing (NMR-QC).^51^ Indeed, besides being an important resource for scalability, this coupling can play a key role in specific protocols such as quantum-error correction.^25,43^ Moreover, it leads to large splittings between nuclear levels, making the thermal initialization in a pure state possible at mK temperatures. Finally, the unparalleled degree of tailoring of the spin Hamiltonian of magnetic molecules^60^ is a crucial advantage with respect to standard NMR-QC systems.

The next steps will involve the addition of higher-frequency pulses to control also electronic degrees of freedom, e.g., to mimic the interaction with a heat bath and then simulating open quantum systems.^49,61^ Moreover, the use of more levels and/or multispin molecules will largely extend the class of Hamiltonians addressable by our quantum simulator.

## Experimental Section

### Synthesis

A single crystal of isotopically enriched ^173^Yb(trensal) diluted at 1% into the isotructural Lu(trensal) was grown according to a published method for Er(trensal),^62^ where instead of using Er(OTf)_3_·9H_2_O as in the published method, ^173^Yb(OTf)_3_·9H_2_O and Lu(OTf)_3_·9H_2_O in the molar ratio 1:99 were used. Both Ln salts were synthesized according to a literature procedure, where the corresponding Ln_2_O_3_ was dissolved in boiling dilute triflic acid, and the Ln salt was obtained by slow evaporation of the corresponding solution.^63^ Isotopically enriched ^173^Yb_2_O_3_ was obtained from Neonest AB. Inductively coupled plasma mass spectrometry (ICP–MS) was used to determine the dilution of ^173^Yb(trensal) in Lu(trensal). ICP–MS was performed at the Department of Chemistry, University of Copenhagen on a Bruker Aurora Elite. Small crystals of ^173^Yb_0.01_Lu_0.99_(trensal) grown in the same tube as the one used for the experiments in the main text were dissolved in boiling nitric acid (14%). The nitric acid was prepared by diluting TraceSelect grade conc. nitric acid with Milli-Q water. The solution was then diluted with TraceSelect grade nitric acid (2%) until the concentration of ^173^Yb and Lu were within the calibration range of the instrument (1–50 ng/mL). Prior to determining the concentrations of ^173^Yb and Lu, the ICP–MS instrument was tuned using six standard solutions with concentrations of Yb and Lu spanning the range 0–50 ng/mL. These standard solutions were prepared by diluting a reference solution from Inorganic Ventures using TraceSelect grade nitric acid (2%). For the measurements of the Yb concentration, the instrument was programmed only to detect the ^173^Yb isotope. The ICP–MS measurement afforded a ratio of 9:991 ^173^Yb/Lu.

### Apparatus

The experimental apparatus for the characterization and control of the nuclear qudit has been specifically designed by combining the potentialities of the homemade broadband NMR spectrometer “HyReSpect”^64^ with a fast state-of-the-art arbitrary waveform generator (Arb Rider AWG-5062**D**, hereafter AWG) from Active Technologies. The multifrequency pulse sequences for the coherent manipulation of the nuclear qudit were in fact generated by the AWG externally triggered by the spectrometer, while the spectrometer was devoted to the final state detection. The characteristics of the experimental setup are particularly suitable for the present experiment: a flat response over a wide frequency span, very short dead times (<1.3 μs) to make echo-detection compatible with the qudit phase memory time, fast RF switching, a broadband receiver stage, and fast signal averaging.

The high sensitivity of the technique, enhanced by the strong hyperfine interactions of ^173^Yb(trensal), allows for the use of a NMR probe covering a wide frequency range (±30 MHz in our experiments), which can be attained by inserting a parallel resistor in the LC circuit. The loss in sensitivity  due to the diminished *Q*-factor of the probe was compensated by the isotopic enrichment of the target ^173^Yb species.

### Calibration

Rabi nutation experiments on each transition *f*_η_ were performed by implementing a (θ(*t*))_η_ – (π)_η_ echo sequence, where the first pulse of variable length induces the nutation of the spin system in the rotating frame, while the refocusing is generated by the π-pulse. The decay observed in the intensity of Rabi oscillation (see inset of Figure 1d) is dominated by the inhomogeneity of the driving field *B*_1_, which adds to the 1/*T*_2_^η^ rate.

Relaxation times *T*_1_^η^ between each pair of levels were measured by exploiting a double-frequency sequence generated by the AWG, of the type . Indeed, the sequence to measure the time *T*_1_^η^ (corresponding to the transition *f*_η_: |η – 1⟩ ↔ |η⟩) consists of (i) a population transfer to one of the two targeted nuclear states induced by π-pulse on a neighboring transition *f*_η±1_ and (ii) the detection of the increment of the Hanh-echo signal on *f*_η_ due to the induced out-of-equilibrium surplus population. The variable delay τ enables the determination of time required for the recovery of the thermal state populations on the targeted nuclear states |η – 1⟩ and |η⟩, i.e., *T*_1_^η^. The *T*_1_^η^ decays are then subtracted by the Hahn-echo initial amplitude of the transition used for the detection. Studying the relaxation of a nuclear state population transferred to a nearby state under these conditions is different from the standard inversion-recovery method, since here the effect of the relaxation is probed on a transition not directly affected by the first excitation pulse. This provides a lower bound to the relaxation time of each pair of nuclear spin levels.

Single-quantum coherence times *T*_2_^η^ were measured by a standard  Hanh-echo sequence, exploiting the standard spectrometer setup. The measurement of the multiple-quantum coherences required instead a multifrequency pulse sequence generated by the AWG, for the preparation of the desired double- or triple-coherent superposition of states by addressing only consecutive transitions. The sequence for generating the double-quantum coherences can be written as (π/2)_η+1_ – (π)_η+2_ – τ – (−π)_η+2_. First, a coherent superposition  is created between consecutive states by addressing the transition *f*_η+1_. A π-pulse on *f*_η+2_ is then used to implement a state-swap between |η + 1⟩ and |η + 2⟩, yielding the desired double-quantum coherent superposition . After a variable delay τ to follow the coherence decay, a (−π) pulse on *f*_η+2_ is implemented to back-swap the states. This final step recovers the now-decayed single-quantum coherent superposition on *f*_η+1_, which can be detected by the spectrometer. For triple-quantum coherences, an additional (π)_3_ pulse (together with the corresponding back-swap (−π)_3_ one) is needed in order to prepare the  coherent superposition.

Multiple-quantum coherences were then measured by exploiting a  detection sequence, where the first pulse was generated by the AWG and the last one by the spectrometer (hence only the latter was phase-coherent with the detection reference). The spin coherence induced by the first  pulse, which would appear in principle as a (not observable) spin echo, is also encoded by this pulse into population differences. Such a longitudinally encoded frozen-in replica of the phase coherence present after the first pulse is then turned into transverse coherence by the second  pulse and then detected by the spectrometer as a spin echo^a^. The same detection method was used to measure the decay of the coherences induced by the pseudopurification sequences, to check that they are completely lost after the waiting time ∼2.5*T*_2_^η^ before starting the quantum simulation (see Figures S5 and S7). For the quantum simulation of the transverse field Ising model, the pseudopure state was prepared with a  sequence. The first π pulse induces a state-swap between |2⟩ and |3⟩, followed by the  on *f*_2_ creating a superposition between states |1⟩ and |2⟩ with equal amplitudes. Given the very similar Boltzmann population differences of the three involved levels, this sequence yields (apart from a contribution proportional to identity and a scale factor) a dominant population in |0⟩ (0.75), small populations in |1⟩ (0.11) and |2⟩ (0.14). This enabled us to test the simulation starting from a non-trivial initial state.

Quantum simulations were performed with an oscillating field *B*_1_ ∼ 1 G and *B*_1_ ∼ 5 G for the QTM Hamiltonian and for the TIM model, respectively. In the latter case, we have used shorter pulses (larger *B*_1_) because the pulse sequence is much longer. Moreover, the pulse duration has been tuned independently for each transition, in order to match their durations and compensate the effects of the broad-resonance of the NMR probe. A higher static field (*B*_0_ = 0.22 T) has been chosen for the TIM model quantum simulation to amplify the coherence time, given the much longer duration of the pulse sequence.

All the detected echoes were then Fourier-transformed, phase-corrected, and analyzed in the frequency domain by picking the spectral amplitude of the echo at a fixed frequency shift.

### Observables

The Hahn echo sequences at the end of the quantum simulations measure the differences between the populations of neighboring levels of the hardware *P*_η_ – *P*_η+1_. From these quantities, it is possible to extract physical observables, mapped on the hardware state population.

For the QTM problem, the target magnetization of the spin *S* = 1 is given by ⟨*S*_*z*_⟩ = *P*_1_ – *P*_–1_. By mapping the target states |*M* = 1⟩, |*M* = 0⟩, |*M* = −1⟩ into the hardware states |0⟩, |1⟩, |2⟩, the observable becomes ⟨*S*_*z*_⟩ = *P*_0_ – *P*_2_. Since the only accessible quantities are the population difference between neighbor nuclear states, the magnetization of the simulated system (Figure 2f) can be rewritten in terms of quantities that can be directly extracted from the experiment
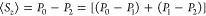
6For the TIM model, the target magnetization of the two-spin system is analogously defined as ⟨*S*_*z*_⟩ = *P*_↑↑_ – *P*_↓↓_. Again, by mapping the target states |↑↑⟩, |↑↓⟩, |↓↑⟩, |↓↓⟩ into the hardware states |0⟩, |1⟩, |2⟩, |3⟩, the observable becomes ⟨*S*_*z*_⟩ = *P*_0_ – *P*_3_. Thus, the magnetization of the simulated system (Figure 4a), in terms of experimentally accessible quantities, becomes
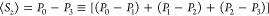
7For this Hamiltonian, we have also extracted the equal-time correlation ⟨*s*_*z*1_*s*_*z*2_⟩. This can be extracted by exploiting completeness relations as follows
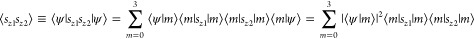
8where we have labeled by |*m*⟩ = |0⟩, |1⟩, |2⟩, |3⟩ the hardware eigenstates and exploited the fact that the target observables *s*_*z*1_ and *s*_*z*2_ are diagonal on this basis. By finally noting that
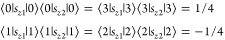
9and rewriting |⟨ψ|*m*⟩|^2^ = *P*_*m*_, we get
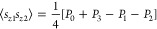
10

### Numerical Calculation

Numerical calculations to reproduce the implemented quantum simulations have been performed by solving the Lindbald master equation
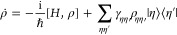
11where ρ is the system density matrix in the eigenbasis, ρ = ∑_ηη′_ρ_ηη′_|η⟩⟨η′|, *H* = *H*_0_ + *H*_1_(*t*) is the system Hamiltonian (including time-dependent pulses), and γ_ηη′_ are pure dephasing rates of each specific superposition between eigenstates |η⟩ and |η′⟩. In the reported experiments, |η⟩ ≈ |*m*_*S*_, *m*_*I*_⟩ and we have focused on the subspace with fixed *m*_*S*_ = 1/2. Hence, rates γ_ηη′_ between states with different *m*_*I*_ correspond to the inverse of the single and multiple-quantum coherence times discussed in the main text. Additional mechanisms depending on the details of the setup, like inhomogeneities of the driving fields, could contribute to γ_ηη′_. These additional dephasing rates have been determined in the quantum tunneling experiment from the observed damping of the oscillations and included in the corresponding calculations. Conversely, to pinpoint the effect of decoherence in the complex dynamics associated with the TIM model, only the measured *T*_2_^η^ (single- and multiquantum) have been included in the calculations. The detection procedure has also been simulated. We have found that here pure dephasing acts practically as an overall scaling factor on the measured signal. Hence, we have rescaled both signal and calculations to the known value at *t* = 0.

### Sequence Optimization

The quantum simulation of the TIM model [target Hamiltonian 4] involves a Suzuki–Trotter decomposition, in which rotations of the target qubits are alternated to an entangling *ZZ* evolution *U*_*ZZ*_(*J*τ) = exp[−i*s*_*z*1_*s*_*z*2_*J*τ], τ = *t*/*n*. In order to reduce the number of pulses to be subsequently implemented, we have exploited the following identity
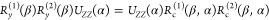
12where *R*_*y*_^(*i*)^(β) = exp[−i*s*_*yi*_β], *R*_c_^(1)^(β) = *R*_α_^(1)^(β) ⊗ |0⟩⟨0| + *R*_–α_^(1)^(β) ⊗ |1⟩⟨1| and *R*_α_(β) = exp[−i(cos α*s*_*y*_ – sin α*s*_*x*_)β]. Analogous expressions hold for *R*_c_^(2)^(β). In practice, this corresponds to including *U*_*ZZ*_ in the subsequent planar rotation. The rotation axis in the plane (α) corresponds to the phase factor of the pulse. Note that the effect of the entangling *U*_*ZZ*_ gate is still present, because *R*_c_^(*i*)^ are conditional (entangling) gates in the two-qubit basis of the target system.

## Supporting Information Available

NMR spectra, calibration results, and additional measurements of relaxation and coherence times (PDF)

## Footnotes

a This process is referred to in the NMR literature as a “stimulated echo”,^65^ whose textbook sequence consists of three  pulses: the first  pulse creating the coherence between |η⟩ and |η + 1⟩ and the other two  pulses representing the detection sequence, where the last pulse “stimulates” an extra spin echo. We stress that, in our experiment, the detected signal cannot be due to either the trivial Hahn echo of the two  pulses themselves, nor any other combination of pulses generated by the AWG alone. Since the spectrometer and the AWG are mutually incoherent, such spin echoes would average out on signal accumulation. On the contrary, reciprocal coherence of the two instruments is not needed if spin coherence is first encoded in populations, as sketched in the main text.
