# Supplementary material for: Proof-of-Concept Quantum Simulator Based on Molecular Spin Qudits
Source: J Am Chem Soc. 2023 Dec 26;146(1):1053–61. doi: 10.1021/jacs.3c12008 (PMC10785809; doi:10.1021/jacs.3c12008)
Supplement: Supplementary file 1 — ja3c12008_si_001.pdf [file ja3c12008_si_001.pdf]

Proof-of-concept Quantum Simulator based on Molecular Spin Qudits

Supporting Information

Simone Chicco, Giuseppe Allodi,* Alessandro Chiesa,* Elena Garlatti,* Christian D. Buch, Paolo Santini, Roberto De Renzi, Stergios Piligkos, and Stefano Carretta

1. Multiple-qubits or Single-qudit

In a quantum information perspective, multi-spin molecules can be more properly described either as multiple qubits or as a single qudit, depending on the hierarchy between the different interactions in the spin Hamiltonian.

A multi-qubit quantum computing register is properly defined on a collection of weakly interacting spins ½. In this regime the Hamiltonian eigenstates are practically factorized and can thus be used to encode the states of the computational basis. Then, quantum gates between these states are driven by external tools, such as electro-magnetic pulses, which can turn on and off the inter-qubit coupling only when required by a given algorithm.

Conversely, strongly coupled spins often give rise to entangled eigenstates, which can be exploited to define the states of a single qudit.

This also gives a chemical intuition of the two opposite regimes: molecules characterized by weak couplings (compared to differences of Zeeman energies) provide a good multi-qubit register, while strongly interacting spins (typically transition metal ions) can encode an individual qudit. Examples of the first class are the Ln-trimers of Refs. [1,2] or chains of Cr_7Ni qubits linked by long bridges in a significant applied field.[3,4] Paradigmatic examples of qudits are instead single-molecule magnets, where the exchange coupling between different spins is dominant. Indeed, Mn_{12} and Fe_8 were already proposed as qubits for the implementation of the Grover's algorithm in 2001.[5] Molecules containing a single ion with S>½ represent the extreme simplification of this idea, such as the $TbPc_2$ [6,7] or the Yb(trensal) [8] nuclear spin proposed as our simulator.

2. Supplementary Figures and Tables

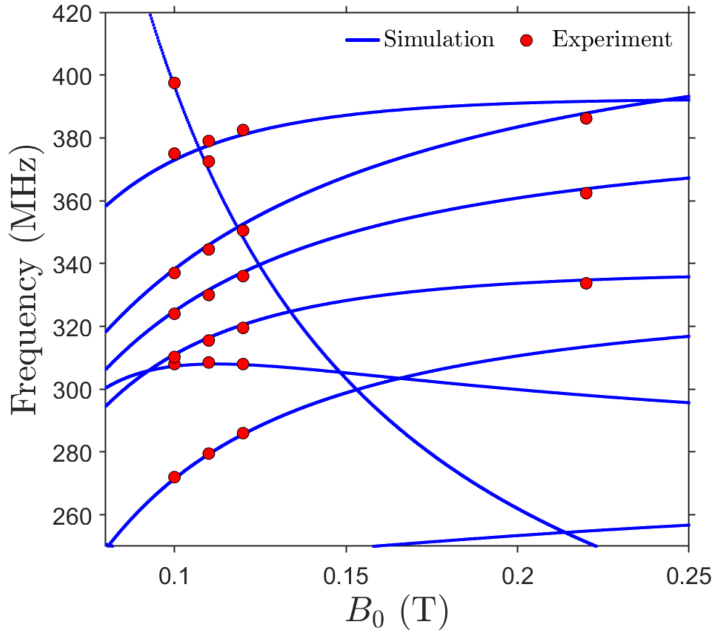

Supplementary Figure 1. NMR transition frequencies vs field. Nuclear transition frequencies as a function of the applied static field (blue line) were calculated to confirm the correct assignment of the detected transitions (red dots) and the parameters of the spin Hamiltonian in Eq.(1) of the main text.

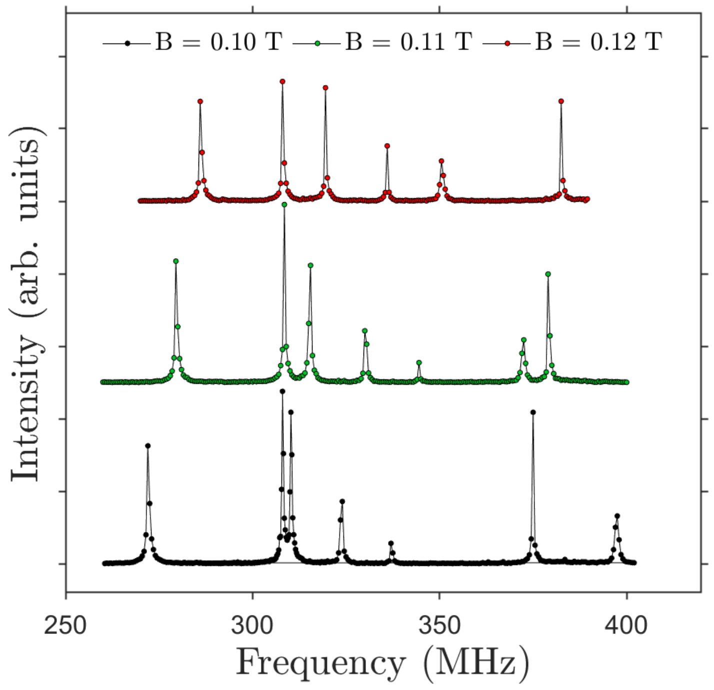

Supplementary Figure 2. NMR spectra. [^{173}Yb(trensal)] NMR spectra at the static fields B_0 = 0.1, 0.11, 0.12 T, measured on a reduced frequency window around the frequencies selected to implement the quantum simulations.

	B_0 = 0.12 T	B_0 = 0.22 T
f_1	750 ns	358 ns
f_2	750 ns	300 ns
f_3		358 ns

Supplementary Table 1. Duration of π Excitation Pulses. Duration of the rf-pulses corresponding to a π rotation at each frequency, obtained by nutation experiments. The pulses calibration is reported for both the working fields of the quantum simulatior.

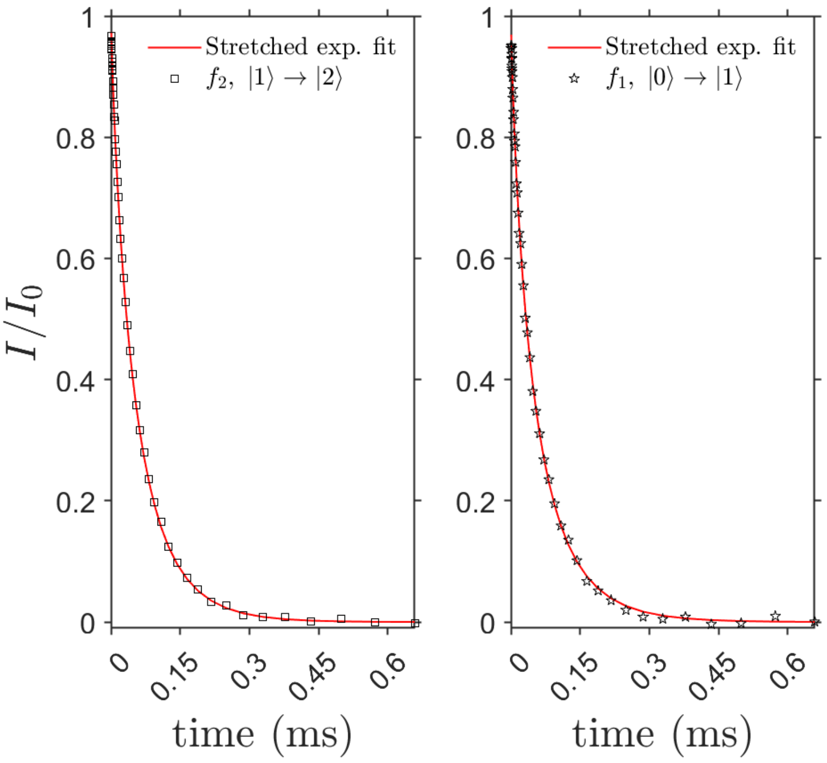

Supplementary Figure 3. Relaxation times at B_0 = 0.12 T. Relaxation times T_1^{η} measured (black squares & stars) on nuclear transitions f_2 (left panel) and f_1 (right panel) of [^{173}Yb(trensal)], with the double-frequency protocol described in the Methods section of the main text, at the applied static field B_0 = 0.12 T. The zero baseline has been re-scaled with the Hahn-echo initial amplitude of the transition used for the detection. Red lines are the stretch-exponential fitting curves, with $T_1^{\eta=2}$=53(3) μs ($T_1^{\eta=1}$ = 52(3) μs) and $\beta_{\eta=2}$=0.85(5) ($\beta_{\eta=1}$=0.85(5)), respectively.

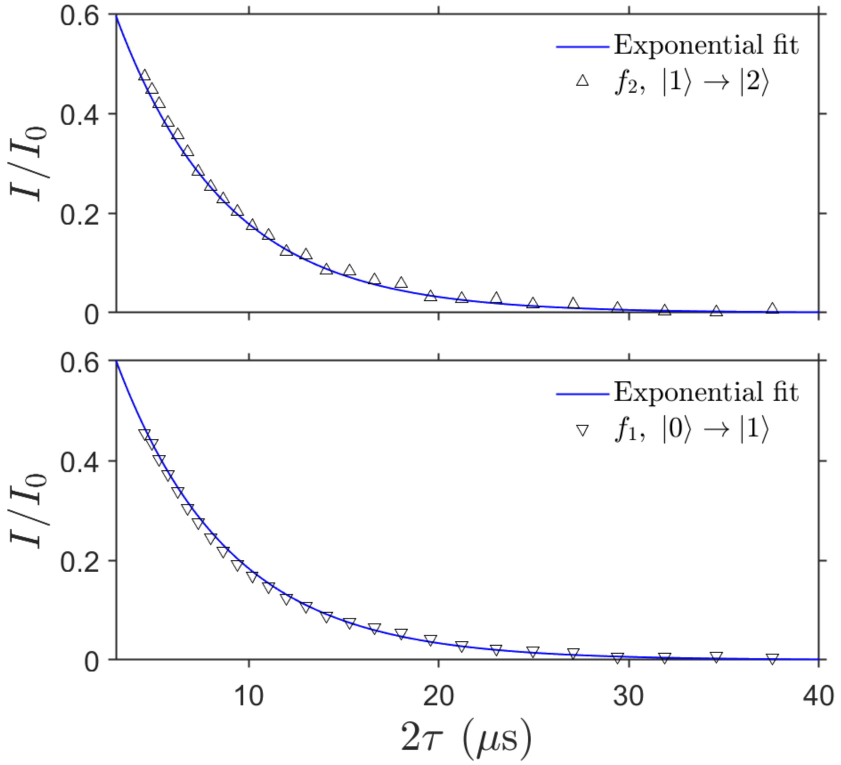

Supplementary Figure 4. Single-quantum coherence times at B_0 = 0.12 T. Single-quantum coherence times T_2^{η} measured (black triangles) on nuclear transitions f_2 (top panel) and f_1 (bottom panel) of [^{173}Yb(trensal)] at the applied static field B_0 = 0.12 T. Blue lines are the single exponential fitting curves with $T_2^{\eta=2}$=5.8(6) µs ($T_2^{\eta=1}$ = 5.9(6) µs), respectively.

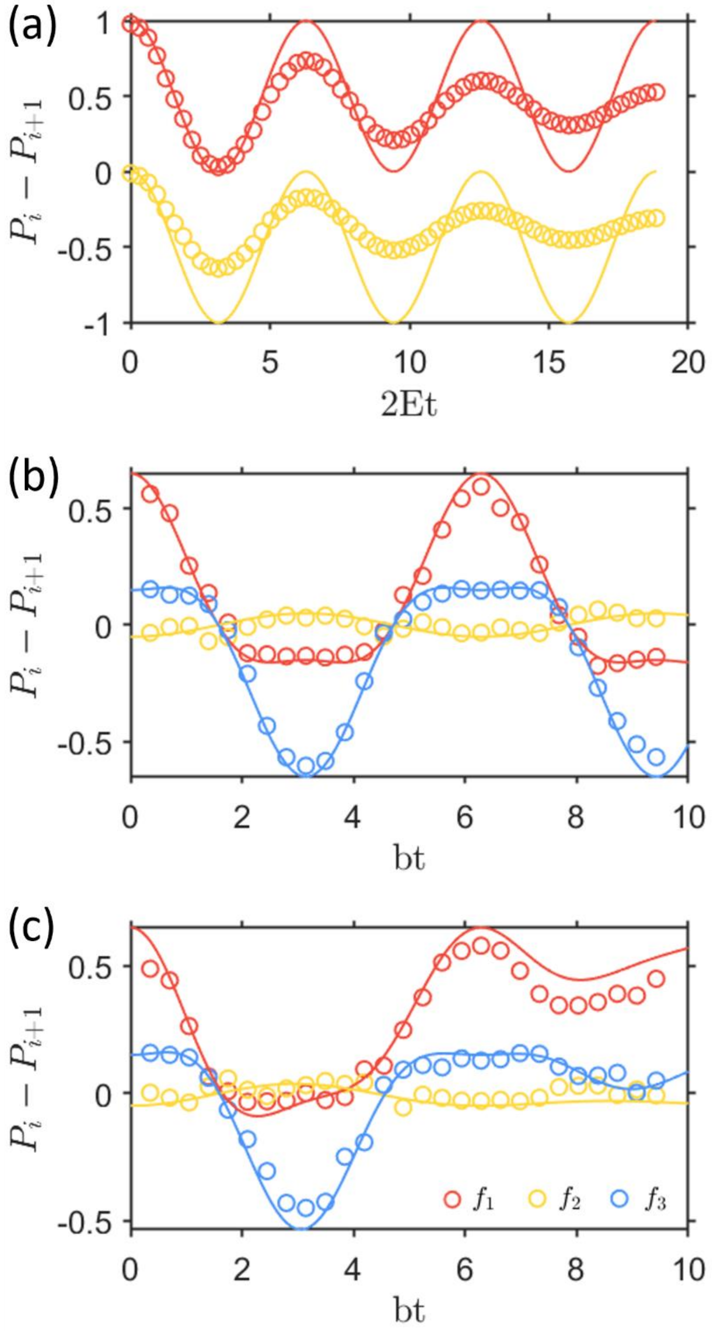

Supplementary Figure 5. Quantum Simulation without multi-quantum decoherence. (a) Calculated Difference of populations between states |0⟩ ↔ |1⟩ (f_1, red) and |1⟩ ↔ |2⟩ (f_2, yellow), at B_0 = 0.12 T, at the end of the quantum simulation of the QTM model. Lines corresponds to noiseless calculations, while circles include the incoherent Lindblad dynamics induced by the measured single-quantum T_2^{η} and the additional dephasing due to inhomogeneities of the driving field. Multi-quantum T_2^{η} are assumed infinite. (b,c) Calculated difference of populations between states |0⟩ ↔ |1⟩ (f_1, red), |1⟩ ↔ |2⟩ (f_2, yellow), and |2⟩ ↔ |3⟩ (f_3, blue), at B_0 = 0.22 T, at the end of the quantum simulation of the non interacting (b) and interacting (c) TIM model. Lines correspond to the noiseless calculation, while circles includes the incoherent Lindblad dynamics induced by the measured single-quantum coherence times. Multi-quantum T_2^{η} are assumed infinite.

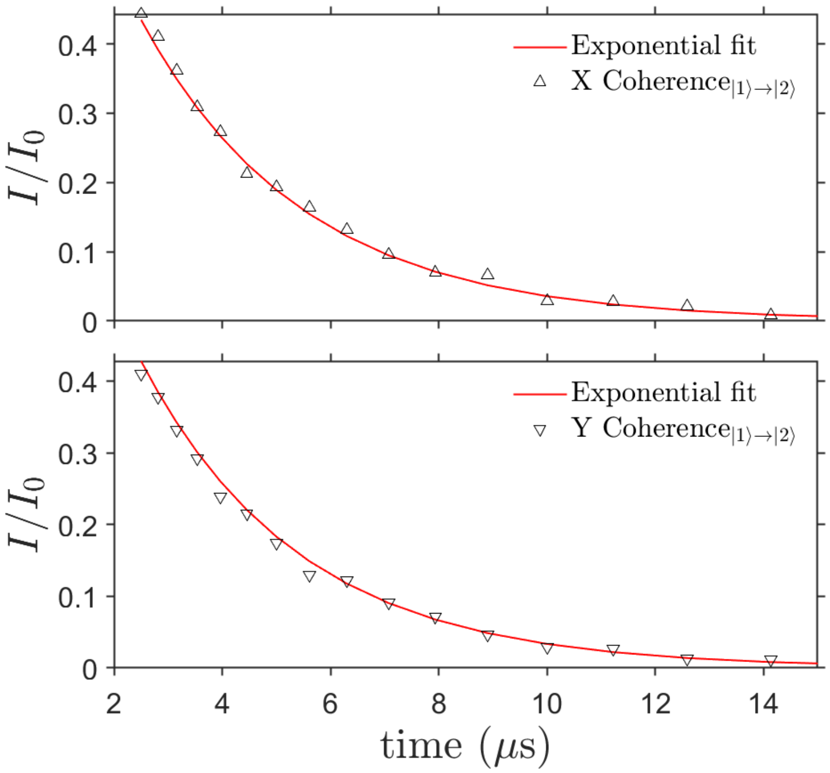

Supplementary Figure 6. Coherences decay after the two-levels purification protocol. Decay of the coherences induced by the two-levels purification protocol, applied before the quantum simulation of the quantum tunnelling Hamiltonian. Measurements were performed with the π/2-π/2 detection sequence explained in the Methods section of the main text. Rotations induced by the first π/2 pulse around the X (top panel) or Y axis (bottom panel) allowed us to probe the decay of the real and imaginary part of the off-diagonal elements of the density matrix, respectively. The measured coherence decay is consistent with the T_2^{η} previously measured.

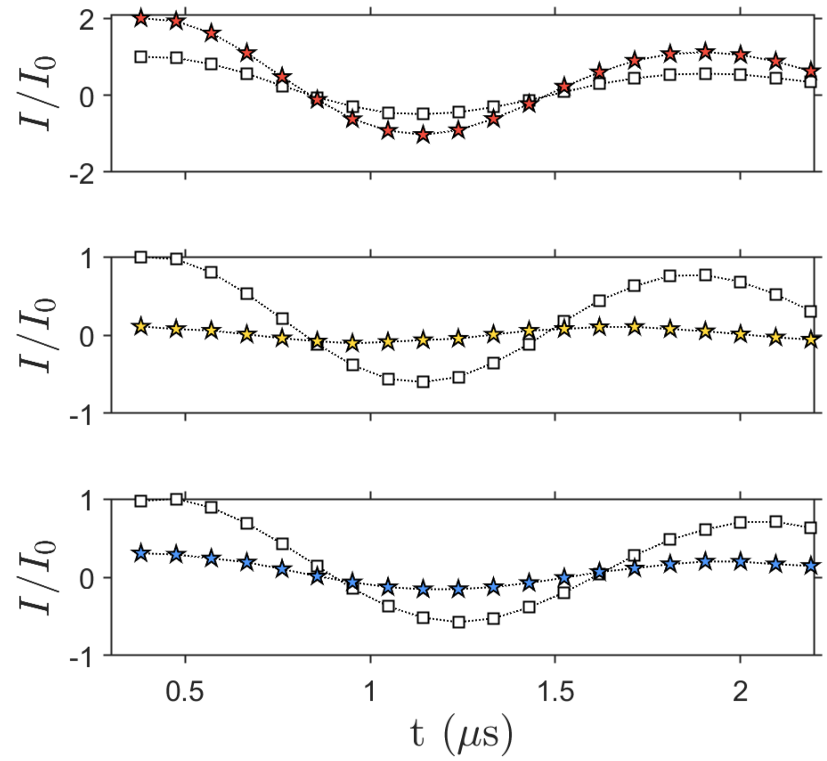

Supplementary Figure 7. Rabi oscillation before and after the three-levels purification protocol. Test of the three-levels purification protocol by sending pulses at frequency f_1, f_2, f_3 (from top to bottom) and comparing the driven dynamics, in preparation of the pseudo-pure states for the quantum simulation of the Transverse Ising model.

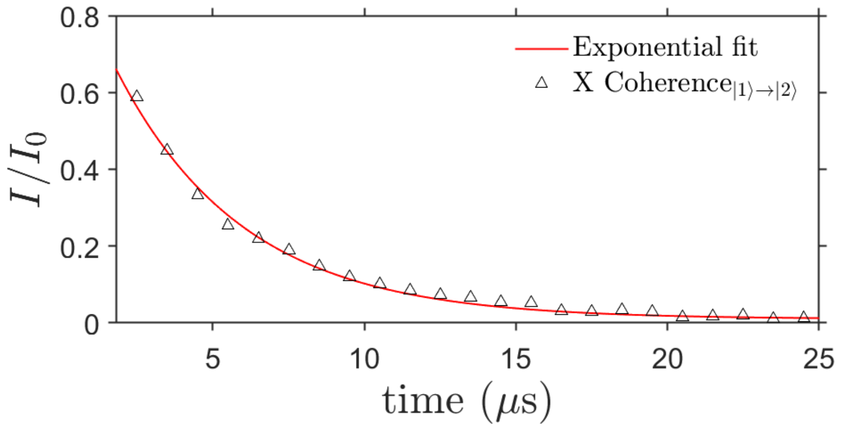

Supplementary Figure 8. Coherences decay after the three-levels purification protocol. Decay of the coherences induced between states $|1\rangle$ and $|2\rangle$ by the three-levels purification protocol, applied before the quantum simulation of the Transverse Ising model. Measurements were performed with the π/2-π/2 detection sequence explained in the Methods section of the main text. The measured coherence decay is consistent with the T_2^{η} previously measured.

REFERENCES

[1] J. Baldoví, S. Cardona-Serra, J. M. Clemente-Juan, L. Escalera-Moreno, A. Gaita-Ariño and G. Mínguez Espallargas, *EPL,* **2015**, 110, 33001.

[2] E. Macaluso, M. Rubín, D. Aguilà, A. Chiesa, L. A. Barrios, J. I. Martínez, P. J. Alonso, O. Roubeau, F. Luis, G. Aromí, S. Carretta, *Chem. Sci.*, **2020**,11, 10337-10343.

[3] S. J. Lockyer, A. Chiesa, A. Brookfield, G. A. Timco, G. F. S. Whitehead, E. J. L. McInnes, S. Carretta, R. E. P. Winpenny, *J. Am. Chem. Soc.* **2022**, 144, 35, 16086–16092.

[4] C. J. Rogers, D. Asthana, A. Brookfield, A. Chiesa, G. A. Timco, D. Collison, L. S. Natrajan, S. Carretta, R. E. P. Winpenny, A. M. Bowen, *Angew. Chem.Int. Ed*. **2022**,61,e202207947.

[5] M. Leuenberger, D. Loss, *Nature*, **2001**, 410, 789–793.

[6] S. Thiele, F. Balestro, R. Ballou, S. Klyatskaya, M. Ruben, W. Wernsdorfer. *Science* **2014**, 344, 1135–1138

[7] E. Moreno-Pineda, C. Godfrin, F. Balestro, W. Wernsdorfer, M. Ruben, *Chem. Soc. Rev.,***2018**,47, 501-513

[8] R. Hussain, G. Allodi, A. Chiesa, E. Garlatti, D. Mitcov, A. Konstantatos, K. Pedersen, R. D. Renzi, S. Piligkos, S. Carretta, *J. Am. Chem. Soc*., **2018**,140, 9814.
